# Oncogene amplification in male breast cancer: analysis by multiplex ligation-dependent probe amplification

**DOI:** 10.1007/s10549-012-2051-3

**Published:** 2012-04-13

**Authors:** Robert Kornegoor, Cathy B. Moelans, Anoek H. J. Verschuur-Maes, Marieke C. H. Hogenes, Peter C. de Bruin, Joost J. Oudejans, Luigi Marchionni, Paul J. van Diest

**Affiliations:** 1 Department of Pathology, University Medical Center Utrecht, Heidelberglaan 100, 3584 CX Utrecht, The Netherlands; 2 Laboratory for Pathology East Netherlands, Burgemeester Edo Bergsmalaan 1, 7512 AD Enschede, The Netherlands; 3 Department of Pathology, St. Antonius Hospital, Koekoekslaan 1, 3435 CM Nieuwegein, The Netherlands; 4 Department of Pathology, Diakonessenhuis, Bosboomstraat 1, 3582 KE Utrecht, The Netherlands; 5 Johns Hopkins University, 1550 Orleans Street, Baltimore, MD USA; 6 Department of Pathology, University Medical Center Utrecht, PO Box 85500, 3508 GA Utrecht, The Netherlands

**Keywords:** Breast cancer, Male, MLPA, Amplification, Copy number, Survival

## Abstract

Gene amplification is an important mechanism for oncogene activation, a crucial step in carcinogenesis. Compared to female breast cancer, little is known on the genetic makeup of male breast cancer, because large series are lacking. Copy number changes of 21 breast cancer related genes were studied in 110 male breast cancers using multiplex ligation-dependent probe amplification. A ratio of >1.3 was regarded indicative for gene copy number gain and a ratio >2.0 for gene amplification. Data were correlated with clinicopathological features, prognosis and 17 genes were compared with a group of female breast cancers. Gene copy number gain of *CCND1*, *TRAF4*, *CDC6* and *MTDH* was seen in >40 % of the male breast cancer cases, with also frequent amplification. The number of genes with copy number gain and several single genes were associated with high grade, but only CCND1 amplification was an independent predictor of adverse survival in Cox regression (*p* = 0.015; hazard ratio 3.0). In unsupervised hierarchical clustering a distinctive group of male breast cancer with poor prognosis (*p* = 0.009; hazard ratio 3.4) was identified, characterized by frequent CCND1, *MTDH*, *CDC6*, *ADAM9*, *TRAF4* and *MYC* copy number gain. Compared to female breast cancers, *EGFR* (*p* = 0.005) and *CCND1* (*p* = 0.041) copy number gain was more often seen in male breast cancer, while copy number gain of *EMSY* (*p* = 0.004) and *CPD* (*p* = 0.001) and amplification in general was less frequent. In conclusion, several female breast cancer genes also seem to be important in male breast carcinogenesis. However, there are also clear differences in copy number changes between male and female breast cancers, pointing toward differences in carcinogenesis between male and female breast cancer and emphasizing the importance of identifying biomarkers and therapeutic agents based on research in male breast cancer. In addition *CCND1* amplification seems to be an independent prognosticator in male breast cancer.

**Electronic supplementary material:**

The online version of this article (doi:10.1007/s10549-012-2051-3) contains supplementary material, which is available to authorized users.

## Introduction

Gene amplification is important in the development and progression of cancer and could serve as a potential biomarker for prognosis or as a target for molecular therapy. In female breast cancer, *HER2* is the best described oncogene with frequent amplificaion. *HER2* amplification is correlated with poor survival and good response to targeted therapy [1, 2]. Other genes, like epidermal growth factor receptor (*EGFR*), Fibroblast growth factor receptor 1 (*FGFR1*), topoisomerase IIa (*TOP2A*) and *MYC* are also involved in female breast cancer and have prognostic and therapeutic implications [3–6].

Compared to female breast cancer, there is yet little knowledge regarding the genetic makeup of male breast cancer, because male breast cancer is a rare disease and the few available studies are based on small single institutional series [7]. Treatment of male breast cancer has largely been extrapolated from its female counterpart, while there are important differences between male and female breast cancer, with higher ratios of estrogen receptor (ER) and progesterone receptor (PR) positivity in men [8–10]. Also the distribution of molecular subtypes by immunohistochemical analysis shows important differences. Luminal type A and B are by far the most frequently encountered subtypes and HER2 driven, basal-like and triple-negative tumors are very rare in men [11, 12]. The few gene expression studies performed recently in men showed that there might be important differences in molecular profile between male and female breast cancer [13–15]. However, the clinical and prognostic significance of genetic alterations in relevant breast cancer genes still needs to be elucidated in male breast cancer.

Multiplex ligation-dependent probe amplification (MLPA) analysis is a high throughput genomic technique enabling relative quantification of copy number or promoter hypermethylation in a variety of genes in one reaction, based on the simultaneous amplification of specifically hybridized probes on DNA that can be derived from paraffin embedded material [16, 17]. We previously showed in female breast cancer that MLPA analysis with a dedicated “breast cancer kit” allows evaluation of copy numbers in 21 important breast cancer genes, providing an overview of the most common amplifications [18]. In the present study, we used MLPA to investigate copy number changes of 21 (female) breast cancer related genes in a large group of male breast cancer and correlate these genomic anomalies with clinicopathological features, patients’ outcome, and with previously obtained MLPA data from female breast cancers.

## Materials and methods

### Patients: specimens and clinical information

All consecutive cases of surgical breast specimens of invasive male breast cancer from 1986 to 2010 were collected from four different pathology labs in The Netherlands (St. Antonius Hospital Nieuwegein, Diakonessenhuis Utrecht, University Medical Center Utrecht, Laboratory for Pathology East Netherlands) as described in more detail previously [12]. Hematoxylin and eosin (HE) slides were reviewed by three experienced observers (PJvD, RK, AM) to confirm the diagnosis and to type and grade according to current standards. Pathology reports were used to retrieve information on age, tumor size, and lymph node status. A total of 110 cases from which the paraffin blocks contained enough tumor for DNA isolation were included. The age of these patients ranged from 32 to 89 years (average: 66 years). Tumor size ranged from 0.8 to 5.5 cm (average: 2.2 cm). In 86 % lymph node status was known and 55 % of these patients had lymph node metastases. The majority of cases were diagnosed (according to the WHO) as invasive ductal carcinoma (90 %). The remaining cases were lobular (*n* = 3), mixed type (ductal/lobular) (*n* = 2), invasive cribriform (*n* = 1), papillary (*n* = 1), mucinous (*n* = 2), invasive micropapillary (*n* = 1) or adenoid cystic carcinomas (*n* = 1). According to the modified Bloom and Richardson score [19] most tumors were grade 2 (41 %) or grade 3 (36 %). Mitotic activity was assessed as before [20] with a mean mitotic index of 11 per 2 mm^2^ (range 0–56). For all cases hormone receptor and *HER2* status were re-assessed as described previously [12]. Tissue microarray (TMA) slides were used for immunohistochemical stainings for ER, PR and chromogenic in situ hybridization (CISH) for HER2 assessment, the latter showing HER2 amplification in only 4/110 cases (4 %)*.* TMA slides were also stained for E-cadherin. Most tumors were ER positive (102/110, 93 %) and PR positivity was also common (71/110; 65 %). Only four cases were E-cadherin negative (three lobular carcinomas and one ductal carcinoma).

### DNA extraction and MLPA analysis

Representative tumor areas were identified in HE stained slides and corresponding tumor areas (at least 1 cm^2^) were dissected with a scalpel from 8 μm paraffin slides [21]. DNA was extracted by overnight incubation in proteinase K (10 mg/ml; Roche, Almere, The Netherlands) at 56 °C. After boiling for 10 min and centrifugation, 5 μl of this DNA solution was used for MLPA analysis. MLPA was performed according the manufacturers’ instructions (MRC Holland, Amsterdam, The Netherlands), using a Veriti^®^ 96-well thermal cycler (Applied Biosystems, Foster City, CA, USA). The P078-B1 kit (MRC Holland), containing 21 breast cancer related genes (Table 1), was used as before [18]. All tests were performed in duplicate. Seven negative references samples (normal breast and blood) were included in each MLPA run. The PCR products were separated by electrophoresis on an ABI 3730 capillary sequencer (Applied Biosystems). Mean probe peaks were used for final gene copy number analysis with Genescan v4.1 (Applied Biosystems) and Coffalyser v9.4 (MRC-Holland) software. Cut-off values were set as before with 1.3–2.0 for gene copy number gain, >2.0 for amplification and <0.7 for lost genes. Values between 0.7 and 1.3 were regarded normal [18, 22].

**Table 1:** Contents of the “breast cancer” MLPA kit P078-B1 (MRC Holland)

Gene	Chrom	Gain (%)	Amp (%)	Loss (%)	Function and clinical relevance
*ESR1*	06q25.1	6	0	3	Transcription factor; under debate [40–42]
*EGFR*	07p11.2	22	1	0	Signal transduction; poor survival [4]
*FGFR1*	08p11.23	29	13	0	Signal transduction; poor survival, tamoxifen resistance [5]
*ADAM9*	08p11.23	39	11	1	Protein metabolism; promotes invasion [38]
*IKBKB*	08p11.21	32	6	0	Signal transduction [43]
*PRDM14*	08q13.3	32	9	0	Transcription regulatory protein; chemoresistance [44]
*MTDH*	08q22.1	49	12	0	Signal transduction; promoting metastases, chemoresistance, poor survival [36]
*MYC*	08q24.21	36	10	0	Transcription factor; poor survival [3]
*CCND1*	11q13.2	46	18	1	Signal transduction; ER positivity, poor survival [35]
*EMSY*	11q13.5	10	2	3	Transcription regulatory protein; poor survival [45]
*CDH1*	16q22.1	6	0	9	Cell adhesion [46]
*TRAF4*	17q11.2	41	4	0	Signal transduction [47]
*CPD*	17q11.2	9	0	0	Protein metabolism [48]
*MED1*	17q21.2	23	4	0	Transcriptional coactivator; ER positivity [49]
*HER2*	17q12	17	4	0	Signal transduction; bad survival; trastuzumab response [2]
*CDC6*	17q21.2	41	4	0	Signal transduction [50]
*TOP2A*	17q21.2	26	2	0	Regulation of the topological status of DNA; poor survival, susceptible for certain chemotherapy [6]
*MAPT*	17q21.31	16	0	0	Microtubule stabilization; chemoresistance (taxanes) [51]
*BIRC5*	17q25.3	27	2	0	Signal transduction; predict distant recurrence [52]
*CCNE1*	19q12	2	0	1	Signal transduction; poor survival [53]
*AURKA*	20q13.31	10	4	12	Signal transduction [54]

For each gene, chromosome location (Chrom), gene copy number gain (Gain; >1.3), amplification (Amp; >2.0), gene loss (Loss; <0.7), function and clinical relevance (in female breast cancer) are shown

### Control female breast cancers

A group of female breast cancer described previously was used to study differences in gene copy number change between male and female breast cancer [18]. This group consists of 104 cases with a mean age of 58 years (range 30–86 years). Tumor size ranged from 0.2 to 6.5 cm (average 2.1 cm) and 46 % of the cases had lymph node metastases. Most cases were diagnosed (according to the WHO) as invasive ductal carcinoma (78 %) or invasive lobular carcinoma (11 %). Mean mitotic activity was 21 per 2 mm^2^ and according to the modified Bloom and Richardson score most tumors were grade 2 (34 %) or grade 3 (45 %). ER positivity was common (69 %, 70/101) and 48 % of the tumors were PR positive (48/101). *HER2* amplification defined by immunohistochemistry and CISH was seen in 19 % of cases (19/102). The same “breast cancer kit” (P078-A1 kit; MRC Holland) was used, but because the gene content of the kit had been updated by the manufacturer in the meanwhile, only 17 genes could be compared between the groups. In addition, for two genes (*EGFR* and *HER2*) one of the probes was modified and for five genes one probe was deleted. Some reference probes were modified as well (Supplementary Table 1).

### Statistics

Statistical calculations were performed using SPSS for Windows v15.0. Correction for multiple comparisons was applied by resetting the 0.05 threshold according to the Holm–Bonferroni method. Differences between gene copy number and clinicopathological characteristics were calculated with ANOVA for continuous variables and with Pearson Chi-square (or Fisher’s exact test when appropriate) for categorical variables. The following clinicopathological features were dichotomized: age (>50 years), tumor size (>2.0 cm), mitotic activity (>8 mitoses/2 mm^2^), and histological grade (grade 1/2 vs 3). Correlation between number of gene amplification and clinicopathological features were calculated with Spearman’s rho. Unsupervised hierarchical clustering using the statistical program R (www.r-project.org) was performed to identify relevant clusters and co-amplification. We used the maximum distance and Ward’s clustering method and calculated the stability of the clusters with pvclust. Logistic regression analysis was performed to compare gene amplification in male and female breast cancer, taking significant differences in clinicopathological features between the two groups into account. Information regarding prognosis and therapy was requested from the Integral Cancer registration The Netherlands (IKNL). Survival data were available for 101 cases with a mean follow up of 5.7 years. Therefore, survival analysis was based on 5 years survival rates. For univariate survival analysis Kaplan–Meier curves were plotted and analyzed with the log rank test. Multivariate survival analysis was done with Cox regression including the variables that were significant in univariate analysis.

## Results

### Copy number analysis by MLPA

**Fig. 1:**
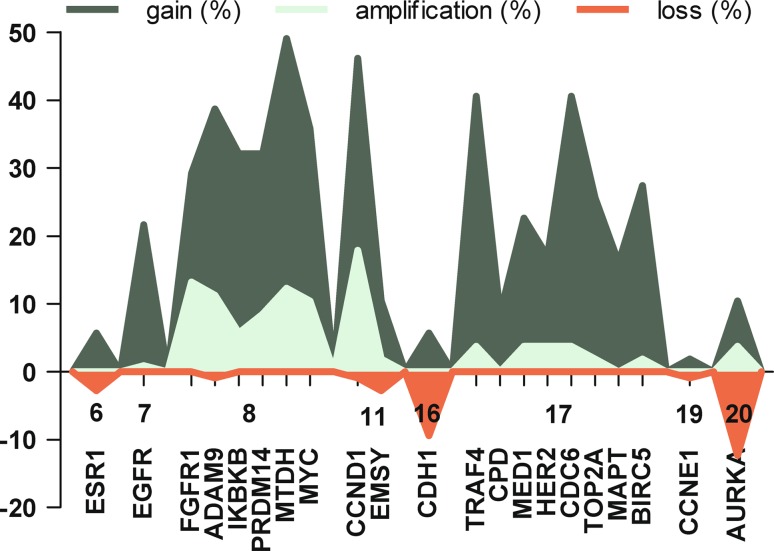
Copy number change of 21 genes with corresponding chromosome in 106 male breast cancer patients. Copy number gain (Gain, >1.3); Amplification (>2.0); Loss (<0.7)

In 4 cases the amount of DNA was insufficient, leaving 106 cases of male breast cancer for further analysis. Gene copy number status of the 21 analyzed genes is presented in Table 1 and Fig. 1. All genes analyzed showed copy number alterations with varying frequencies. The average number of genes with copy number gain was four (range 0–12), of which one (range 0–8) showed amplification.

Copy number gain was most frequently seen in the genes *MTDH* (52/106; 49 %) and *CCND1* (49/106; 46 %), and these genes were also frequently amplified (13/106; 12 % and 19/106; 18 % respectively). The genes analyzed on chromosome 8 (*FGFR1*, *ADAM9*, *IKBKB*, *PRDM14*, *MTDH* and *MYC*) were also frequently affected with high rates of copy number gain and amplification. Thirteen cases (12 %) showed copy number gain of all genes analyzed on chromosome 8. Also the genes located on chromosome 17 were often affected, particularly *TRAF4*, *CDC6*, and *BIRC5* with copy number gain in 37, 37 and 26 % of cases, respectively. However, amplification of these genes was rare (<4 %). Amplification of *HER2* was also rare (4/106; 4 %). In five cases (5 %), all genes analyzed on chromosome 17 showed copy number gain. In 17 % of cases (18/106) no gene copy number changes were found. Losses were rare and seen in only seven genes of which *CDH1* (10/106; 9 %) and *AURKA* (13/106; 12 %) were most frequently affected.

### Correlation with clinicopathological features

Tumors with a copy number gain in one or more genes tended to have a more aggressive phenotype with more mitoses (*p* = 0.004) and a higher histological grade (*p* = 0.007) compared to tumors without gene copy number alterations. The number of genes with copy number gain was significantly correlated with a high mitotic count (*p* = 0.001) and a high histological grade (*p* < 0.001).

Copy number gain in the genes *MED1* (*p* < 0.001), *BIRC5* (*p* < 0.001), *PRDM14* (*p* = 0.003), and *MTDH* (*p* = 0.003) were significantly correlated with high grade male breast cancer. *MED1* and *HER2* copy number gain were significantly correlated with high mitotic count (*p* < 0.001 and *p* = 0.003, respectively). We found trends for other genes, which did not remain significant after correction for multiple comparisons (Table 2).

**Table 2:** Correlation between gene copy number gain (>1.3) and clinicopathological features

Gene	Age (mean) young	Mitoses high (>8)	Mitoses (mean) high	Grade high (3)	LN meta negative	ER negative
*ESR1*						
*EGFR*						0.038
*FGFR1*	0.019					
*ADAM9*	0.043	0.017	0.004			
*IKBKB*						0.033
*PRDM14*		0.049		**0.003**		
*MTDH*		0.019	0.005	**0.003**		
*MYC*				0.023		
*CCND1*			0.010			
*EMSY*				0.016		
*CDH1*						
*TRAF4*						
*CPD*					0.010	
*MED1*		**0.001**	**<0.001**	**<0.001**		
*HER2*		0.025	**0.003**	0.014		
*CDC6*			0.027			
*TOP2A*		0.045	0.025	0.013		
*MAPT*						
*BIRC5*		0.018	0.024	**<0.001**		
*CCNE1*						
*AURKA*						

Tumor size and PR were not correlated with any of the studied genes (not shown). *p* values were calculated with Pearson Chi-square or Fisher’s exact test when appropriate (number of events <5) for categorical variables and ANOVA for continuous variables. Significant *p* values after correction for multiple comparison (Holm–Bonferroni method) are depicted in *bold*. See Supplementary Table 2 for full data

*LN* *meta* lymph node metastases

Three out of the four tumors with *HER2* amplification (defined by CISH) also showed *HER2* amplification using MLPA (*p* < 0.001). Loss of *CDH1* was not correlated with any clinicopathological feature and loss of the *CDH1* gene did not correlate with E-cadherin expression.

### Comparison with female breast cancer

**Fig. 2:**
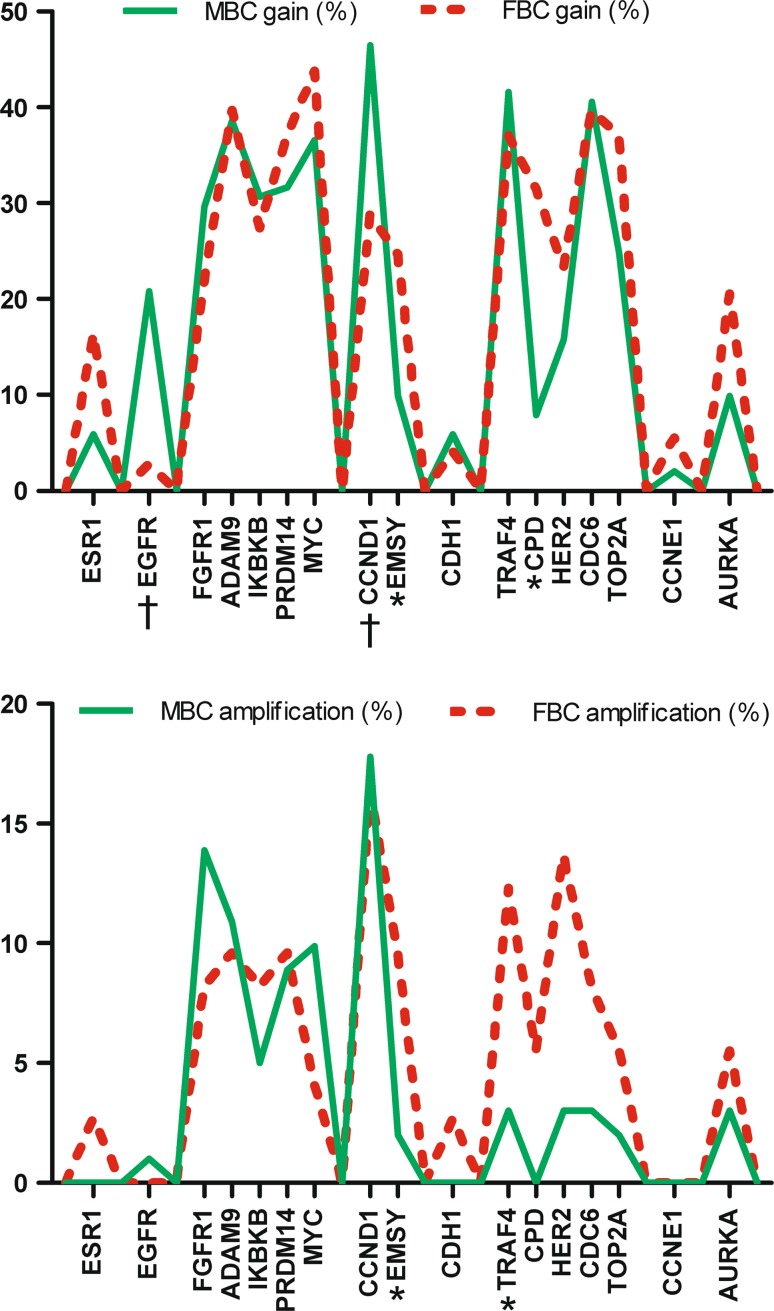
Comparison of frequency of copy number gain (>1.3, *upper graph*) and amplification (>2.0, *lower graph*) of 17 genes between luminal type male and female breast cancer. *MBC* Male breast cancer, *FBC* Female breast cancer, *Amp* amplification. †Genes significantly more affected in men, *genes significantly more affected in women

Because breast cancer is a heterogeneous disease only luminal type male and female breast cancers (defined by ER and/or PR expression) were compared. In this approach mitotic count (11 vs 13 mitoses) and grade (37 vs 33 % grade 3 tumors) were quite similar in male and female breast cancers. Only age was significantly different, as male breast cancer patients were significantly older (*p* < 0.001). Figure 2 illustrates gene copy number gain and gene amplification in 101 male and 73 female breast cancer cases. *EGFR* (*p* = 0.005) and *CCND1* (*p* = 0.041) copy number gain were independent predictors of gender in logistic regression, and these genes were more often gained in male breast cancer. *EMSY* (*p* = 0.004) and *CPD* (*p* = 0.001) copy number gain were also independent predictors of gender and these genes were more frequently gained in female breast cancer. Two genes, *TRAF4* (*p* = 0.024) and *EMSY* (*p* = 0.041) were more often amplified in female breast cancer. None of the studied genes was significantly more frequently amplified in men.

### Cluster analysis

**Fig. 3:**
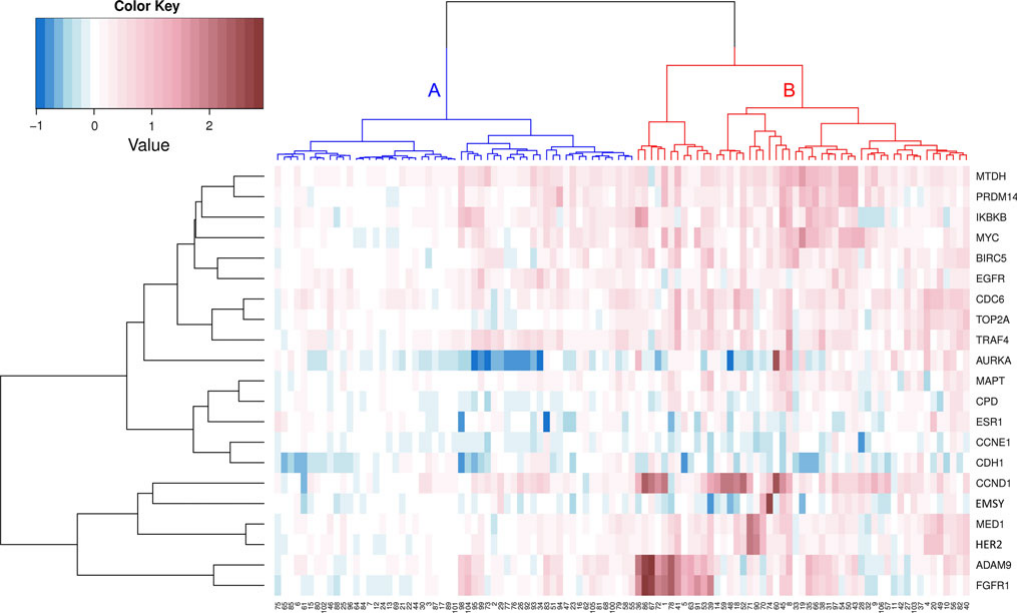
Unsupervised hierarchical clustering of copy number changes in 21 breast cancer related genes in 106 male breast cancer patients. The identified clusters of patients (*horizontal*) are depicted in different colors

Unsupervised hierarchical clustering revealed a separate gene cluster, consisting of *FGFR1*, *ADAM9*, *HER2*, *MED1*, *EMSY*, and *CCND1* (Fig. 3). One small sub-cluster was formed by *FGFR1* and *ADAM9* which showed simultaneously copy number gain in 29 % of all cases (31/106). Gains in both genes was correlated with younger age (62 vs 68 years; *p* = 0.019). No associations with other clinicopathological features were found.

Reasoning from the cases, two major clusters were found (Fig. 3). These clusters were stable according to the approximately unbiased *p* values calculated with pvclust (*p* < 0.001). Cluster A consisted of 55 cases and was characterized by a low rate of gene copy number gain and gene amplification. Cluster B consisted of 51 cases and was characterized by CCND1 (73 %), *MTDH* (69 %), *CDC6* (63 %), *ADAM9* (57 %), *TRAF4* (57 %) and *MYC* (53 %) copy number gain. The male breast cancers in cluster B showed significantly more mitosis compared to the tumors in cluster A (8 vs 14 mitosis; *p* < 0.001). Cluster B tumors were also more often grade 3 (*p* = 0.020) and were larger (2.4 vs 2.0 cm; *p* = 0.036) compared to cluster A tumors.

### Survival analysis

**Fig. 4:**
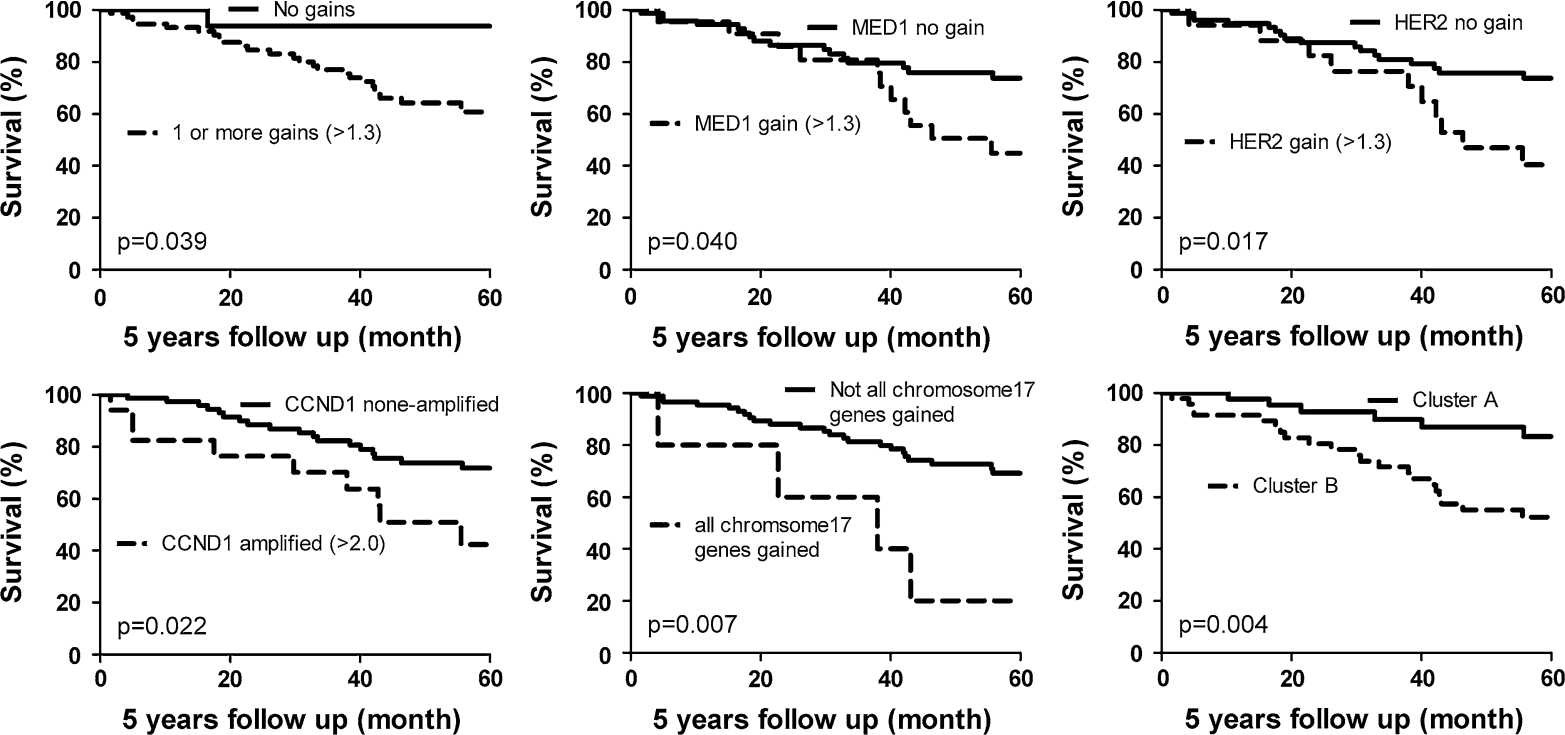
Kaplan–Meier survival curves with corresponding *p* values (log rank) according to 1 or more gained genes, MED1 (>1.3), HER2 (>1.3), CCND1 amplification (>2.0), copy number gain of all analyzed genes located on chromosome 17 and cluster A versus cluster B

Grade 3 (*p* = 0.027), high mitotic count (>8; *p* = 0.015) and large tumor size (>2.0 cm; *p* = 0.036) were correlated with a decreased 5 years survival*.* Chemotherapy was given in 14 % of the cases and 40 % received hormone therapy. Both treatment regimes did not correlate with patients’ survival (*p* = 0.700 and *p* = 0.140, respectively). Univariate survival analysis is presented in Fig. 4. Tumors with one or more gains had a poorer outcome compared with tumors without gains (*p* = 0.039). *MED1* and *HER2* copy number gain also seem to correlate with poor survival (*p* = 0.040 and *p* = 0.017, respectively). In case amplification was analyzed the genes *CCND1* (*p* = 0.022) and *EMSY (p* = 0.040) were correlated with decreased survival. However, for *EMSY* only two cases were amplified. In case correction for multiple comparisons was performed, no single prognostic factor remained significant. On the other hand, tumors with a copy number gain of all genes on chromosome 17 had a poorer survival (*p* = 0.007). Cluster B from unsupervised hierarchical clustering had adverse patients’ outcome (*p* = 0.004).

Using a Cox regression analysis, *CCND1* amplification appeared to be the only single gene which was a predictor of survival aside from grade, mitotic count, and tumor size (*p* = 0.015; hazard ratio 3.0). When chemotherapy and hormone therapy were included in Cox regression, *CCND1* was retained as an independent prognosticator. However, hormone therapy was an independent prognostic factor as well and was correlated with a favorable prognosis (*p* = 0.004; hazard ratio 0.225). Cluster B tumors (*p* = 0.009; hazard ratio 3.4) and tumors with copy number gain of all analyzed genes on chromosome 17 (*p* = 0.005; hazard ratio 4.8) were also independent predictors of poor survival. The multivariate models are supplied in supplementary format (Supplementary Table 3).

## Discussion

Gene amplification is an important mechanism of oncogene activation and is crucial for the development and progression of cancer. The identification of frequent copy number change in certain chromosomal regions can lead to identification of functional important genes in carcinogenesis, reveal distinctive groups of breast cancer and can be used as prognostic markers. Knowledge of gene profiling in male breast cancer is sparse, because male breast cancer is a rare disease and most studies are based on small single institutional series. In the present study we used the high throughput technique MLPA to study gene copy number alterations of 21 breast cancer related genes in a large multi-institutional cohort of 106 male breast cancer patients.

The average amount of genes that showed copy number gain was four (range 0–12), of which one (range 0–8) was amplified. 18 cases (17 %) did not show any copy number change in the studied genes. These 18 cases tended to be low grade cancers with few mitoses and seem to have favorable prognosis compared to male breast cancers with gene copy number gain. The number of genes with copy number gain was correlated with high grade and a high mitotic count. This is in line with female breast cancers, as the genome in high grade female breast cancers is also more rearranged and these patients have a poor outcome [23, 24]. Simultaneous copy number gain of all analyzed genes on chromosome 8, 11, and 17 was seen in 12, 10, and 5 % of the cases, respectively. This points to polysomy or gain of whole chromosome arms, a finding often seen in male breast cancer [14]. This is interesting, as polysomy of e.g., chromosome 17 has been refuted in female breast cancer [25–30]. In our group of male breast cancer copy number gain of all genes located on chromosome 17 was an independent predictor of adverse prognosis.

Using unsupervised hierarchical cluster analysis a small sub-cluster was formed by *FGFR1* and *ADAM9*. In female breast cancer, co-amplification of these chromosomal regions is also a common finding [24, 31]. In addition, two stable clusters of male breast cancer patients were identified with additional prognostic value to classical clinicopathological prognosticators.

*HER2* amplification defined by MLPA in the present study strongly correlated with *HER2* amplification status defined by CISH on TMA slides. Small differences found could be due to heterogeneity of tumors which could be missed or overrepresented in TMA slides. We have also previously validated MPLA against CISH and FISH [16].

*CCND1* and *MTDH* were the genes which most frequently showed copy number gain (49 and 46 %, respectively), and often had amplification, indicating that these genes probably play an important role in male breast carcinogenesis. *CCND1* encodes for cyclin D1, which is a cell cycle protein driving cell cycle progression through the G1 phase. It also enhances ER-mediated gene transcription and is especially overexpressed in ER positive female breast cancer [32]. *CCND1* amplification has been linked to ER positive tumors as well, although some did not find such a correlation [18, 24, 33]. In the present study, we could not identify a correlation between *CCND1* copy number gain or amplification and ER status. A clear cut association between *CCND1* amplification and patients’ outcome in female breast cancer is lacking, but *CCND1* amplification may be associated with a poor prognosis, particularly in ER positive tumors [23, 24, 33–35]. In the present group of male breast cancers, tumors with *CCND1* copy number gain tended to have a higher mean mitotic count compared to tumors without *CCND1* amplification, a finding which is in line with the encoding protein function. More importantly, amplification of *CCND1* was the only single gene which correlated with poor survival and had additional prognostic value aside from tumor size, mitotic count, and histological grade using a Cox regression analysis.

*MTDH* is involved in several signaling pathways and amplification of *MTDH* promotes metastases, enhances chemo-resistance and is associated with poor outcome in female breast cancer patients [36]. In line with these findings in females, we demonstrated that male breast cancer with *MTDH* copy number gain showed a more aggressive phenotype with a high mitotic count and a high histological grade. However, no correlation with prognosis and *MTDH* copy number change was found in our group of male breast cancer and no correlation with lymph node metastasis was found either.

The genes located on different amplicons on chromosome 8p11 (*FGFR1*; 29 %, *ADAM9*; 39 % and *IKBKB*; 32 %) were also often gained. These genes have been correlated with ER positive female breast cancers [18]. Since most male breast cancer cases are ER positive (93 % in the present group), frequent copy number gain of these genes can be explained by the high ratio of ER positive tumors [8, 9]. Nevertheless, we could not confirm the correlation between copy number gain or amplification of these genes and ER positive tumors in male breast cancer. However, in view of the low rate of ER negative male breast cancers in the present study, these results need to be interpreted with care. It is important to note that *FGFR1* amplification enhances tamoxifen resistance, which is particularly clinically relevant in male breast cancer, as endocrine therapy is often indicated in these patients [5]. Since *FGFR1* copy number gain and amplification seems to be common in male breast cancer and is suitable for targeted therapy, this gene could be of further interest in male breast cancer [37]. ADAM9, which is important in cell adhesion and tumor cell invasion, has potential in male breast cancer as well, since this gene is often affected and could be used for targeted therapy [38, 39].

Among the other genes studied, copy number gain of *MED1*, *PRDM14*, and *BIRC5* were associated with a high grade phenotype, indicating that these genes play a role in the development or progression of aggressive male breast cancer. Indeed MED1 copy number gain tends to correlate with poor survival. We could not confirm the prognostic relevance of the other genes in male breast cancer patients.

Comparison with 103 female breast cancers revealed differences in copy number change between male and female breast cancer in a variety of genes, pointing toward differences in carcinogenesis. In male breast cancer *CCND1* and *EGFR* were more often gained than in the female breast cancer group. In the group of female breast cancers *EMSY* and *CPD* copy number gain were seen more often than in males. In line with a previous comparative genomic hybridization study, female breast cancer showed more frequent amplification in a variety of genes, particularly in *TRAF4* and *EMSY* [14]. None of the genes studied were significantly more often amplified in male breast cancer. Alongside gender specific differences between male and female breast cancers, differences in genetic predisposition may also influence the genetic profile of these tumors. Approximately 10 % of men with breast cancer are known to have a genetic predisposition, and especially BRCA2 mutations seem to be important [7]. Differences in BRCA mutations status between male and female breast cancers would have implications for the genetic makeup of these tumors and deserves further investigation.

In conclusion, copy number gain of the genes *CCND1* (11q13), *TRAF4* (17q11), *CDC6* (17q21), and *MTDH* (8q22) is very common in male breast cancer (>40 %) and these genes probably play a role in male breast carcinogenesis. Tumors with copy number gain of one or more genes showed a highly malignant phenotype. Also *MED1*, *PRDM14*, *MTDH*, and *BIRC5* seem to be important in the development or progression of high grade male breast cancer. Amplification of *CCND1* was the most important single gene as it correlated with poor survival and had prognostic value in addition to the classical clinicopathological prognostic factors. Using unsupervised hierarchical clustering a distinctive group of male breast cancer tumors was identified with poor survival. Compared to female breast cancer *CCND1* and *EGFR* were found to be more frequently amplified in male breast cancer, while in females *EMSY* and *CPD* were more often involved and more frequent amplifications of *TRAF4* and *EMSY* were found. Our results point toward important differences in carcinogenesis between male and female breast cancer, emphasizing the importance in identifying specific biomarkers and therapeutic targets for male breast cancer.

## Electronic supplementary material

Below is the link to the electronic supplementary material.

Supplementary material 1 (DOC 119 kb)

Supplementary material 2 (DOC 144 kb)

Supplementary material 3 (DOC 54 kb)
