# Supplementary material for: Oncogene amplification in male breast cancer: analysis by multiplex ligation-dependent probe amplification
Source: Breast Cancer Res Treat. 2012 Apr 13;135(1):49–58. doi: 10.1007/s10549-012-2051-3 (PMC3413821; doi:10.1007/s10549-012-2051-3)
Supplement: Supplementary file 1 — Supplementary material 1 (DOC 119 kb) [file 10549_2012_2051_MOESM1_ESM.doc]

**Supplemental Table 1** Contents of the “breast cancer” MLPA kit P078-B1 breast tumor (MRC Holland; lot 0109) and modification compared to MLPA kit P078-A1.

**Table 1a** Contents of the MLPA kit P078-B1. Modified probes are depicted in red. New genes are depicted in green.

| **Gene** | **Probe** | **Chromosome posistion** | **Map View** | **Length (nt)** |
| --- | --- | --- | --- | --- |
| ESR1 | 11998-L12826 | 06q25.1 | 06-152.457215 | 232 |
| ESR1 | 11996-L12824 | 06q25.1 | 06-152.423838 | 214 |
|  |  |  |  |  |
| EGFR | 05969-L05386 | 07p11.2 | 07-055.233957 | 265 |
| EGFR | 02063-L03283 | 07p11.2 | 07-055.191055 | 427 |
|  |  |  |  |  |
| FGFR1 | 01046-L00624 | 08p11.23 | 08-038.434092 | 373 |
| FGFR1 | 04440-L03826 | 08p11.23 | 08-038.391533 | 400 |
| ADAM9 | 11992-L12820 | 08p11.23 | 08-038.998319 | 136 |
| IKBKB | 11993-L12821 | 08p11.21 | 08-042.292902 | 148 |
| IKBKB | 12003-L12831 | 08p11.21 | 08-042.302676 | 454 |
|  |  |  |  |  |
| PRDM14 | 12002-L12830 | 08q13.3 | 08-071.130073 | 445 |
| MTDH | 04151-L03506 | 08q22.1 | 08-098.742504 | 281 |
| MTDH | 04152-L03507 | 08q22.1 | 08-098.788082 | 337 |
| MYC | S0247-L08464 | 08q24.21 | 08-128.821796 | 118 |
| MYC | 00580-L00625 | 08q24.21 | 08-128.822151 | 157 |
| MYC | 00672-L00169 | 08q24.21 | 08-128.822001 | 238 |
|  |  |  |  |  |
| CCND1 | 00583-L00148 | 11q13.2 | 11-069.175089 | 292 |
| CCND1 | 05402-L04808 | 11q13.2 | 11-069.167779 | 463 |
| EMSY | 09173-L09347 | 11q13.5 | 11-075.902087 | 132 |
| EMSY | 09175-L09349 | 11q13.5 | 11-075.926543 | 256 |
|  |  |  |  |  |
| CDH1 | 02410-L02237 | 16q22.1 | 16-067.404826 | 178 |
| CDH1 | 02860-L01849 | 16q22.1 | 16-067.328716 | 355 |
|  |  |  |  |  |
| TRAF4 | 09176-L09350 | 17q11.2 | 17-024.098403 | 124 |
| CPD | 09628-L09913 | 17q11.2 | 17-025.795018 | 226 |
| MED1 | 09963-L13205 | 17q21.2 | 17-034.840858 | 346 |
| HER2 | S0393-L12911 | 17q12 | 17-035.133169 | 113 |
| HER2 | 00675-L00146 | 17q12 | 17-035.118101 | 142 |
| HER2 | 12048-L12913 | 17q12 | 17-035.136344 | 244 |
| HER2 | 00986-L00406 | 17q12 | 17-035.127183 | 310 |
| CDC6 | 08611-L13204 | 17q21.2 | 17-035.699283 | 196 |
| TOP2A | 11994-L12822 | 17q21.2 | 17-035.818297 | 172 |
| TOP2A | 11999-L13177 | 17q21.2 | 17-035.812698 | 329 |
| TOP2A | 12000-L12828 | 17q21.2 | 17-035.816651 | 364 |
| MAPT | 08358-L08211 | 17q21.31 | 17-041.423085 | 416 |
| BIRC5 | 03717-L02410 | 17q25.3 | 17-073.722036 | 316 |
| BIRC5 | 03025-L14708 | 17q25.3 | 17-073.724340 | 383 |
| BIRC5 | 03189-L02540 | 17q25.3 | 17-073.722396 | 436 |
|  |  |  |  |  |
| CCNE1 | 02881-L02348 | 19q12 | 19-035.005214 | 166 |
| CCNE1 | 09170-L09344 | 19q12 | 19-035.000150 | 190 |
|  |  |  |  |  |
| AURKA | 10236-L10717 | 20q13.31 | 20-054.389980 | 481 |

**Table 1b** Two probes in the P078-A1 breast kit that were modified in the updated P078-B1kit (see table 1a, depicted in red).

| **Gene** | **Probe** | **Chromosome position** | **Map View** | **Length (nt)** |
| --- | --- | --- | --- | --- |
| EGFR | 05959-L05376 | 07p11 | 07-055.196767 | 247 |
| HER2 | 00717-L00390 | 17q12 | 17-035.136627 | 337 |

**Table 1c** Five probes in the P078-A1 breast kit, not present anymore in the updated P078-B1 kit.

| **Gene** | **Probe** | **Chromosome position** | **Map View** | **Length (nt)** |
| --- | --- | --- | --- | --- |
| CCND1 | 00601-L00162 | 11q13 | 06-152.307247 | 184 |
| HER2 | 12044-L12908 | 17q12 | 07-055.196767 | 268 |
| CCNE1 | 05782-L05724 | 19q12 | 11-069.165399 | 391 |
| CDH1 | 02414-L01860 | 16q22 | 17-035.122165 | 283 |
| ESR1 | 12001-L12829 | 06q25 | 16-067.419579 | 436 |

**Table 1d** Reference probes in the P078-B1 breast kit. Modified genes are depicted in red. Three probes were not used as reference probes (depicted in blue), because of common copy number change in these probes.

| **Gene** | **Probe** | **Chromosome position** | **Map View** | **Length (nt)** |
| --- | --- | --- | --- | --- |
| PLA2G6 | 09570-L10024 | 22q13 | 22-036.865968 | 220 |
| IDH3A | 00978-L00565 | 15q25 | 15-076.239491 | 274 |
| ANXA7 | 00971-L09490 | 10q22 | 10-074.828036 | 301 |
| RTN4 | 00963-L00550 | 02p16 | 02-055.068269 | 408 |
| VWF | 11350-L12075 | 12p13 | 12-006.015847 | 184 |
| SLITRK3 | 10223-L10704 | 03q26 | 03-166.390414 | 208 |
| TRPM3 | 10224-L10705 | 09q21 | 09-072.566364 | 490 |
| RELN | 10218-L14675 | 07q22 | 07-102.864424 | 500 |
|  |  |  |  |  |
| ZNF198 | 05730-L06767 | 13q11 | 13-019.465729 | 202 |
| GLRA1 | 08964-L09059 | 05q33 | 05-151.214886 | 391 |
| LPIN2 | 09205-L09581 | 18p11 | 18-002.950707 | 472 |

**Table 1e** Reference probes in the P078-A1 kit. Modified genes are depicted in red (not present in the updated P078-B1 kit).

| **Gene** | **Probe** | **Chromosome position** | **Map View** | **Length (nt)** |
| --- | --- | --- | --- | --- |
| PLA2G6 | 09570-L10024 | 22q13 | 22-036.865968 | 220 |
| IDH3A | 00978-L00565 | 15q25 | 15-076.239491 | 274 |
| ANXA7 | 00971-L09490 | 10q22 | 10-074.828036 | 301 |
| RTN4 | 00963-L00550 | 02p16 | 02-055.068269 | 408 |
| LPIN2 | 09205-L09581 | 18p11 | 18-002.950707 | 472 |
| NP220 | 00992-L00552 | 02p13 | 02-071.430713 | 202 |
| CASP2 | 02051-L01583 | 07q35 | 07-142.699658 | 319 |
| TSPAN15 | 00973-L00560 | 10q22 | 10-070.936627 | 382 |
