# Supplementary material for: Oncogene amplification in male breast cancer: analysis by multiplex ligation-dependent probe amplification
Source: Breast Cancer Res Treat. 2012 Apr 13;135(1):49–58. doi: 10.1007/s10549-012-2051-3 (PMC3413821; doi:10.1007/s10549-012-2051-3)
Supplement: Supplementary file 2 — Supplementary material 2 (DOC 144 kb) [file 10549_2012_2051_MOESM2_ESM.doc]

**Supplemental Table 2** Frequencies of gene copy number gain (>1.3; Table 2a), gene amplification (>2.0; Table 2b) and gene loss (<0.7; Table 2c) according to clinicopathological features. For the continues variables (age and mitoses) mean values are depicted for each gene.

**Table 2a**  Copy number gain

| **Gene** | **age (mean)** | **Mitoses** | **Mitoses (mean)** | **Grade** | **LN meta** | **ER** |
| --- | --- | --- | --- | --- | --- | --- |
|  |  | **High (>8)** |  | **High (3)** | **negative** | **negative** |
| *ESR1* | 63 | 5 (83%) | 14 | 4 (67%) | 3 (60%) | 0 |
| *EGFR* | 67 | 13 (57%) | 11 | 10 (44%) | 9 (45%) | 4 (17%) |
| *FGFR1* | 62 | 20 (65%) | 12 | 14 (45%) | 16 (57%) | 1 (3%) |
| *ADAM9* | 63 | 28 (68%) | 14 | 19 (46%) | 19 (54%) | 2 (5%) |
| *IKBKB* | 65 | 19 (56%) | 11 | 13 (38%) | 17 (59%) | 5 (15%) |
| *PRDM14* | 67 | 23 (68%) | 12 | 19 (56%) | 15 (54%) | 3 (9%) |
| *MTDH* | 65 | 34 (65%) | 13 | 26 (50%) | 21 (49%) | 4 (8%) |
| *MYC* | 64 | 24 (63%) | 12 | 19 (50%) | 17 (53%) | 1 (3%) |
| *CCND1* | 66 | 29 (59%) | 13 | 21 (43%) | 20 (49%) | 3 (6%) |
| *EMSY* | 67 | 8 (73%) | 14 | 8 (73%) | 3 (30%) | 1 (9%) |
| *CDH1* | 69 | 5 (83%) | 16 | 3 (50%) | 3 (60%) | 0 |
| *TRAF4* | 66 | 28 (65%) | 12 | 20 (47%) | 15 (42%) | 3 (7%) |
| *CPD* | 62 | 5 (56%) | 10 | 4 (44%) | 8 (89%) | 1 (11%) |
| *MED1* | 64 | 20 (83%) | 16 | 16 (67%) | 11 (52%) | 3 (13%) |
| *HER2* | 65 | 14 (78%) | 16 | 11 (61%) | 10 (63%) | 2 (11%) |
| *CDC6* | 67 | 28 (65%) | 13 | 20 (47%) | 21 (54%) | 3 (7%) |
| *TOP2A* | 65 | 19 (70%) | 14 | 15 (56%) | 12 (52%) | 2 (7%) |
| *MAPT* | 68 | 10 (59%) | 12 | 9 (53%) | 9 (60%) | 1 (6%) |
| *BIRC5* | 65 | 21 (72%) | 14 | 20 (69%) | 11 (46%) | 1 (3%) |
| *CCNE1* | 70 | 2 (100%) | 17 | 1 (50%) | 2 (100%) | 0 |
| *AURKA* | 61 | 6 (55%) | 11 | 3 (27%) | 7 (70%) | 1 (9%) |

**Table 2b** Gene amplification

| **Gene** | **age (mean)** | **Mitoses** | **Mitoses (mean)** | **Grade** | **LN meta** | **ER** |
| --- | --- | --- | --- | --- | --- | --- |
|  |  | **High (>8)** |  | **High (3)** | **negative** | **negative** |
| *ESR1* |  |  |  |  |  |  |
| *EGFR* | 79 | 0 | 1 | 0 | 1 (100%) | 0 |
| *FGFR1* | 57 | 8 (57%) | 11 | 6 (43%) | 9 (69%) | 0 |
| *ADAM9* | 57 | 6 (50%) | 10 | 4 (33%) | 9 (82%) | 1 (8%) |
| *IKBKB* | 53 | 2 (33%) | 8 | 2 (33%) | 5 (83%) | 1 (17%) |
| *PRDM14* | 65 | 7 (78%) | 13 | 6 (67%) | 4 (44%) | 0 |
| *MTDH* | 64 | 10 (77%) | 14 | 7 (54%) | 7 (58%) | 1 (8%) |
| *MYC* | 61 | 8 (73%) | 12 | 7 (64%) | 5 (46%) | 1 (9%) |
| *CCND1* | 63 | 13 (68%) | 17 | 8 (42%) | 9 (60%) | 2 (11%) |
| *EMSY* | 65 | 2 (100%) | 18 | 2 (100%) | 0 | 0 |
| *CDH1* |  |  |  |  |  |  |
| *TRAF4* | 60 | 2 (50%) | 8 | 1 (25%) | 2 (50%) | 1 (25%) |
| *CPD* |  |  |  |  |  |  |
| *MED1* | 60 | 4 (100%) | 30 | 4 (100%) | 1 (33%) | 1 (25%) |
| *HER2* | 60 | 4 (100%) | 30 | 4 (100%) | 1 (33%) | 1 (25%) |
| *CDC6* | 63 | 3 (75%) | 11 | 1 (25%) | 3 (75%) | 1 (25%) |
| *TOP2A* | 62 | 1 (50%) | 10 | 1 (50%) | 2 (100%) | 0 |
| *MAPT* |  |  |  |  |  |  |
| *BIRC5* | 74 | 1 (50%) | 10 | 1 (50%) | 1 (50%) | 0 |
| *CCNE1* |  |  |  |  |  |  |
| *AURKA* | 66 | 1 (25%) | 11 | 0 | 2 (50%) | 1 (25%) |

**Table 2c** Gene loss.

| **Gene** | **age (mean)** | **Mitoses** | **Mitoses (mean)** | **Grade** | **LN meta** | **ER** |
| --- | --- | --- | --- | --- | --- | --- |
|  |  | **High (>8)** |  | **High (3)** | **negative** | **negative** |
| *ESR1* | 63 | 2 (67%) | 14 | 0 | 3 (100%) | 0 |
| *EGFR* |  |  |  |  |  |  |
| *FGFR1* |  |  |  |  |  |  |
| *ADAM9* | 63 | 1 (100%) | 14 | 1 (100%) | 0 | 0 |
| *IKBKB* |  |  |  |  |  |  |
| *PRDM14* |  |  |  |  |  |  |
| *MTDH* |  |  |  |  |  |  |
| *MYC* |  |  |  |  |  |  |
| *CCND1* | 66 | 0 | 13 | 0 | 0 | 0 |
| *EMSY* | 67 | 0 | 14 | 0 | 2 (100%) | 1 (33%) |
| *CDH1* | 69 | 3 (30%) | 16 | 1 (10%) | 5 (50%) | 0 |
| *TRAF4* |  |  |  |  |  |  |
| *CPD* |  |  |  |  |  |  |
| *MED1* |  |  |  |  |  |  |
| *HER2* |  |  |  |  |  |  |
| *CDC6* |  |  |  |  |  |  |
| *TOP2A* |  |  |  |  |  |  |
| *MAPT* |  |  |  |  |  |  |
| *BIRC5* |  |  |  |  |  |  |
| *CCNE1* | 70 | 1 (100%) | 17 | 1 (100%) | 0 | 0 |
| *AURKA* | 61 | 6 (46%) | 11 | 5 (39%) | 3 (30%) | 1 (8%) |
