# Supplementary material for: Oncogene amplification in male breast cancer: analysis by multiplex ligation-dependent probe amplification
Source: Breast Cancer Res Treat. 2012 Apr 13;135(1):49–58. doi: 10.1007/s10549-012-2051-3 (PMC3413821; doi:10.1007/s10549-012-2051-3)
Supplement: Supplementary file 3 — Supplementary material 3 (DOC 54 kb) [file 10549_2012_2051_MOESM3_ESM.doc]

**Supplemental Table 3** Multivariate analysis (Cox regression).

**Table 3a** Cox regression model with mitoses (>8), tumor size (>2.0 cm), grade (1/2 versus 3), *CCND1* amplification, *EMSY* amplification, *HER2* copy number gain, *MED1* copy number gain, Hormone therapy and chemotherapy.

| Significant features | | B | SE | Wald | df | Sig. | Exp(B) |
| --- | --- | --- | --- | --- | --- | --- | --- |
|  | Mitoses | ,810 | ,450 | 3,236 | 1 | ,072 | 2,247 |
|  | Size | 1,285 | ,475 | 7,313 | 1 | ,007 | 3,615 |
|  | Hormone therapy | -1,490 | ,510 | 8,520 | 1 | ,004 | ,225 |
|  | *CCND1* amplification | ,955 | ,443 | 4,649 | 1 | ,031 | 2,599 |

**Table 3b** Cox regression model with mitoses (>8), tumor size (>2.0 cm), grade (1/2 versus 3), CCND1 amplification, EMSY amplification, HER2 copy number gain and MED1 copy number gain.

| Significant features | | B | SE | Wald | df | Sig. | Exp(B) |
| --- | --- | --- | --- | --- | --- | --- | --- |
|  | Grade | ,972 | ,409 | 5,657 | 1 | ,017 | 2,643 |
|  | Size | 1,146 | ,463 | 6,130 | 1 | ,013 | 3,147 |
|  | *CCDN1* amplification | 1,095 | ,452 | 5,877 | 1 | ,015 | 2,990 |

**Table 3c** Cox regression model with mitoses (>8), tumor size (>2.0 cm), grade (1/2 versus 3) and Cluster B.

| Significant features | | B | SE | Wald | df | Sig. | Exp(B) |
| --- | --- | --- | --- | --- | --- | --- | --- |
|  | Grade | ,723 | ,398 | 3,309 | 1 | ,069 | 2,061 |
|  | Size | 1,074 | ,448 | 5,749 | 1 | ,016 | 2,928 |
|  | Cluster B | 1,227 | ,470 | 6,814 | 1 | ,009 | 3,412 |

**Table 3d Cox regression model with mitoses (>8), tumor size (>2.0 cm), grade (1/2 versus 3) and copy number gain of all analyzed chromosome 17 genes**

| Significant features | | B | SE | Wald | df | Sig. | Exp(B) |
| --- | --- | --- | --- | --- | --- | --- | --- |
|  | Mitoses | ,927 | ,445 | 4,347 | 1 | ,037 | 2,528 |
|  | Size | ,896 | ,449 | 3,985 | 1 | ,046 | 2,450 |
|  | Copy number gain all chromosome 17 genes | 1,559 | ,556 | 7,851 | 1 | ,005 | 4,753 |
